# A Reflection of Metabolic Syndrome through the Window of COVID-19

**DOI:** 10.3390/vaccines10111966

**Published:** 2022-11-19

**Authors:** Liam Pock Ho, Chuen Wen Tan, Heng Joo Ng, Wai Mun Jason Chay, Jing Yuan Tan, Su Yen Goh

**Affiliations:** 1 Department of Clinical Pathology, Singapore General Hospital, Singapore 169856, Singapore; 2 Department of Hematology, Singapore General Hospital, Singapore 169856, Singapore; 3 Department of Endocrinology, Singapore General Hospital, Singapore 169856, Singapore

**Keywords:** COVID-19, metabolic syndrome, immunity, endothelium, gastrointestinal, mitochondria, micronutrients

## Abstract

COVID-19 and metabolic syndrome, though seemingly different disorders, appear to share certain common pathogenic components, especially in the development of COVID-19-associated diabetes mellitus. The similarities include impairment in immunoendothelial, gastrointestinal, pancreatic, adipose and mitochondrial functions, with several critical micronutrients undergirding the intricate interactions among these dysfunctions. This discussion aims to highlight the parallels between COVID-19 and metabolic syndrome and to propose the possibility of SARS-CoV-2 being a prototype of an acquired etiological agent which can eventually lead to the development of classical metabolic syndrome. Based on the proposed model, the discussion will include the implication for early management of COVID-19 and metabolic syndrome.

## 1. Introduction

COVID-19, an unprecedented pandemic which swept across the globe and in a short span of time, caused innumerable death and great loss to the entire world. However, amidst this unparalleled catastrophe, a glimmer of hope is emerging with improved understanding of COVID-19 and available therapeutic agents. As more information is being gathered for COVID-19, rather unexpectedly, the data are also beginning to converge on another human pandemic, i.e., metabolic syndrome. Early on in COVID-19, three important observations were made which related COVID-19 with metabolic syndrome. Firstly, COVID-19 patients with metabolic diseases including diabetes, obesity or hypertension were shown to be associated with worse outcome [1]. Secondly, COVID-19 could result in the development of a new onset of diabetes in patients without prior history [2]. Thirdly, COVID-19 patients who suffered intestinal dysbiosis were likewise associated with worst clinical outcome [3]. Taken together, they pointed to a circular inter-relationship between COVID-19 and metabolic syndrome, in which either disease can adversely affect the other, and also prompted the possibility that perhaps a unifying pathophysiology may unite all these observations.

A recent concise review on metabolic syndrome discussed various diagnostic criteria and distilled them into four central defining features, i.e., (1) visceral obesity, (2) insulin resistance, (3) atherogenic dyslipidemia and (4) endothelial dysfunction [4]. The author further described intricate interactions of key pathogenic mechanisms, including the production of adipokines which modulates pro-inflammatory cytokines, and the activation of the renin angiotensin system in adipose tissue, which can lead to hypertension and insulin resistance and in turn supports lipolysis to increase free fatty acids, triglyceride and very low-density lipoproteins and causes atherogenic dyslipidemia, while together with hyperglycemia and advanced glycated products can result in endothelial dysfunction, leading to diminished blood flow to skeletal muscle and creating a vicious cycle where endothelial dysfunction in turn worsens insulin resistance [4].

Although these complex interactions seem to defy a straightforward paradigm, upon careful scrutiny of these inter-relationships, a few factors nevertheless appear to emerge as critical in connecting this complicated network, including the key elements of an indeterminate etiological agent, a dysregulated/impaired immune response, as well as endothelial, gastrointestinal and mitochondrial dysfunction. It is with this perspective that we have structured the remaining discussion, i.e., firstly to delineate the connection between viral infection in COVID-19 and metabolic syndrome, and next to describe the association of immune–endothelial dysfunction in COVID-19 and metabolic syndrome, followed by the relationship of essential micronutrients in COVID-19 and metabolic syndrome, before finally bringing everything together into a proposed model.

## 2. Viral Diseases, COVID-19 and Metabolic Syndrome

### 2.1. SARS-CoV-2 Infection in COVID-19

New-onset diabetes in COVID-19 was first described in early 2020, when the authors observed increased frequency of diabetes in COVID-19 patients [2]. Other subsequent reports also supported this initial observation, with one study estimating an increased incident rate ratio for type 2 diabetes of 1.28 (95% CI 1.05–1.57) after SARS-CoV-2 infection [5]. Several pathogenic mechanisms were proposed to account for this connection, and one of the probable driving mechanisms is attributed to the increased infectivity of SARS-CoV-2 within the vascular compartment, secondary to the high affinity of SARS-CoV-2 RBD (receptor-binding domain) for ACE2 (angiotensin-converting enzyme 2) expressed on endothelial cells [6]. After gaining access into the vascular space, SARS-CoV-2 will trigger macrophage activation and immune reaction with increased risk of immunothrombosis and endothelial dysfunction [7]. If uncontained by the local immune response, SARS-CoV-2 can travel via the peripheral blood to infect multiple other organs, and the pancreas, adipose tissue and gastrointestinal tract deserve special attention.

#### 2.1.1. Effect of SARS-CoV-2 Infection in Pancreatic Tissue

SARS-CoV-2 infectivity of the pancreas was confirmed in post mortem tissues, with co-localization of SARS-CoV-2 demonstrated in pancreatic endocrine and exocrine cells [8]. SARS-CoV-2 infects human pancreatic β cells and has been shown to attenuate pancreatic insulin levels and secretion and induce β cell apoptosis, resulting in β cell impairment [9]. In addition, β cells were shown to undergo cellular transdifferentiation with reduced expression of insulin and higher expression, e.g., glucagon and trypsin1 [10]. In COVID-19 patients without any pre-existing history of diagnosis of diabetes, other studies demonstrated abnormalities in glycometabolic control, insulin resistance and beta cell function [11].

#### 2.1.2. Effect of SARS-CoV-2 Infection in Adipose Tissue

SARS-CoV-2 can infect adipocytes, trigger adipose tissue dysfunction to drive insulin resistance and cause hyperglycemia, independent of glucocorticoid treatment [12]. SARS-CoV-2 can elicit an inflammatory response, detectable in COVID-19 autopsy cases associated with inflammatory infiltrate, which may explain the link between obesity and severe COVID-19 [13]. Another important consequence of SARS-CoV-2 infection is the ability to highjack mitochondria and impair mitochondrial dynamics, leading to cell death [14].

#### 2.1.3. Effect of SARS-CoV-2 Infection in Gastrointestinal Tract

SARS-CoV-2 infection causes changes to the gut microbiota, including overall decline in microbial diversity, enrichment of opportunistic pathogens and depletion of beneficial commensals, leading to gut microbiome dysbiosis [15]. Three main pathogenic mechanisms include intestinal inflammation leading to alteration of the gut microbiome, intestinal ACE2 dysregulation inducing gut dysbiosis, and direct infection of microbial bacteria driving gut dysbiosis [16]. Gut microbiota composition is concordant with disease severity and dysfunctional immune responses in COVID-19, which were depleted in gut bacteria with immunomodulatory potential, e.g., Faecalibacterium prausnitzil and several bifidobacteria species. One of the earlier clinical studies demonstrated that 48.5% of COVID-19 patients presented with digestive symptoms as chief complaints, and their prognosis is worse than patients without digestive symptoms [3]. In light of the importance of gastrointestinal function in COVID-19, modulating the gut microbiome may be an effective therapeutic modality in improving clinical outcome. These include modulation with probiotics, prebiotics, diet or nutrient supplementation to prevent gut barrier defects, as well as fecal microbiota transplantation to restore intestinal microbial balance.

### 2.2. Viral Infection in Metabolic Syndrome

Viral infections have long been considered as candidates for environmental triggers of metabolic syndrome, but this idea remains non-conclusive to date. They may play a role on multiple levels, including the generation of inflammation, triggering autoimmunity and promoting beta cell dysfunction and stress as part of a vicious cycle. In view of a possible viral etiology, some on-going trials are currently testing the effect of vaccinations and/or antiviral drugs in treatment of type 1 diabetes [17]. Vaccination as a strategy to treat metabolic syndrome can potentially be double-edged, depending on the residual parenchymal function when the immune response is elicited. Vaccination can be beneficial if given early when beta cell function is still adequate and eradication of the virus will prevent further damages; however, if the immune response is elicited late when there is already significant beta cell dysfunction, this may tip the condition to complete pancreatic failure as a result of collateral damage.

#### 2.2.1. Effect of Viral Infection in Pancreatic Tissue

After infecting the pancreas, viral agents can negatively impact its ability to produce insulin and contribute to the loss of pancreatic β cell function [18]. In addition to β cell impairment, pancreatic exocrine deficiency may also have an important role in pathogenesis in diabetes. It has been reported to be common, with a prevalence ranging from 25–74% in type 1 diabetes and 28–54% in type 2 diabetes [19]. Exocrine pancreatic insufficiency is characterized by loss of exocrine pancreatic enzymes, resulting in an inability to properly digest fats, carbohydrates and proteins. Extrapancreatic sources of gastric lipase and lingual lipase can compensate but are unable to fully restore lipase activity, while protein and carbohydrate digestion can still be maintained by other sources of amylase and protease from salivary, gastric and small intestine tissue [20], thus overall effect results in fat malabsorption syndrome.

#### 2.2.2. Effect of Viral Infection in Gastrointestinal Tract

Microbiota exert important influence on mitochondrial function, e.g., pathogens such as Fusobacterium and Veillonella are shown to control mitochondrial activity in favor of infection and inflammation, while commensal gut microbiota can favorably influence mitochondrial functions related to energy production, mitochondrial biogenesis, redox balance and inflammatory cascades. Under stressful conditions, e.g., viral infection, mitochondria can modulate immune responses, leading to heightened inflammation and resulting in microbiota dysbiosis [21]. Viral infection has been shown to be an instigator of intestinal dysbiosis, leading to type 1 diabetes [22]. Some studies have implicated hepatitis C in type 2 diabetes, with Coxsackievirus B1 and enteroviral infection in type 1 diabetes [17,22,23]. Another study which compared the fecal samples of 11 cases who developed serum autoantibodies associated with type 1 diabetes with controls found that the case subjects with less diversity in intestinal viromes and changes in intestinal virome preceded autoimmunity in this cohort [24].

#### 2.2.3. Effect of Viral Infection in Cardiovascular Disease and Obesity

Different viral infections have been associated with hypertension and cardiovascular disease, e.g., Coxsackievirus, cytomegalovirus, hepatitis C, influenza, MERS-CoV and SARS-CoV and CoV-2, and they may exert adverse outcomes via activation of the renin–angiotensin–aldosterone system (RAAS), induction of inflammatory responses and/or direct organ damages, e.g., endothelial and heart tissue [25]. In fact, in many viral syndromes, endothelial dysfunction is a central component with microvascular thrombosis and endothelitis in multi-organ disease [26]. Likewise, adenovirus Ad36 was reported to be linked with human obesity and has the strongest evidence in the role of obesity. Adenovirus can induce reduction in lipolysis, promote adipogenesis and chronic inflammation and affect lipid metabolism [27]. A study of the association of anti-ADV36-Ab and 25(OH) D in obese children versus normal weight control children demonstrated that the chance of an increase in weight is decreased by 5% for each unit increase in 25(OH) D concentration (OR = 0.95, *p* = 0.012), thus suggested that ADV36-induced lipogenesis may be mediated by vitamin D deficiency in children with obesity [28]. Infectobesity, known as obesity caused by an infectious agent, has opened a new area of research to design and develop new strategies for the treatment of causative agents, by developing pathogen-specific vaccines, antimicrobial therapeutics and control of their spread in geographical regions [27].

## 3. Immuno-Vasculopathy in COVID-19 and Metabolic Syndrome

### 3.1. Immuno-Vasculopathy in COVID-19

After SARS-CoV-2 infection, antibody seroconversion is usually detectable from day 6 and peaks by day 14 [29], which temporally matches the onset of acute respiratory distress in COVID-19, reported to occur between day 6 and day 12 with a median of day 8 from the initiation of symptoms [30].

#### 3.1.1. Hyperinflammation and Defective Regulatory T Cells

Studies on immune reaction in COVID-19 have supported a direct correlation between hyperinflammatory response and severe disease outcome, as evidenced by higher expression of pro-inflammatory cytokines and SARS-CoV-2-specific immunoglobulins, in severe as compared to mild cases [29,31]. Since it is excessive rather than absent/impaired immunological response which is associated with severe outcome, this observation may indicate a defective regulation of immunological reaction involving insufficient or dysfunctional regulatory T (Treg) upstream, which is supported by another study which demonstrated impairment of FOXP3-mediated negative feedback in CD4 + T cells in severe COVID-19 [32].

#### 3.1.2. Paradox of Excessive Response but Worst Viral Clearance

In the presence of excessive response in patients with severe disease, it is unusual that their virus load is higher and persists longer in the respiratory tissue. The median duration of SARS-CoV-2 in the respiratory samples of severe cases was significantly longer than mild cases: 21 days versus 14 days, respectively, *p* = 0.04 [31]. This paradoxical higher immune response with poorer viral clearance may be related to the unique trait of COVID-19, i.e., higher affinity of severe acute respiratory syndrome coronavirus 2 (SARS-CoV-2) RBD for ACE2 expressed on endothelial cells, which increases infectivity within the vascular compartment, and when coupled with significant macrophage/monocyte activation, will trigger coagulation pathways, generating severe microvascular thrombosis as demonstrated in the lungs in COVID-19 autopsies. These thrombosed vascular channels will impair subsequent immune access to the site of infection and prevent further viral clearance, thus accounting for the paradox.

#### 3.1.3. Immunometabolism and COVID-19

The immune system is closely linked to metabolic changes, and there is a dynamic, ongoing crosstalk that exists between immune and metabolism regulatory systems, and these interactions are termed “immunometabolism” [33]. Studies demonstrated that pro-inflammatory cytokines including IL-6, IL-1 or TNF-a can redirect the metabolic pathways in favor of glycolysis over oxidative phosphorylation in mitochondria [34,35]. Since in COVID-19, severe disease is associated with cytokine storm, i.e., higher than normal production of pro-inflammatory cytokines, hyperglycolysis is likely to play an important role in mediating the worst outcome [36].

### 3.2. Immuno-Vasculopathy in Metabolic Syndrome

Immune status in adipose, pancreas and gastrointestinal tissue has been shown to play an important role in the pathogenesis of metabolic syndrome. Chronic low-grade inflammation in adipose tissue [37] activates a type 1 immune response, resulting in production of pro-inflammatory cytokines, e.g., TNF, IFNγ and IL6, and antagonizing Treg functions, leading to obesity and insulin resistance in type 2 diabetes [18,38].

#### 3.2.1. Role of Tregs in Metabolic Syndrome

Tregs were shown to accumulate preferentially in the visceral adipose tissue, and their abundance drops in the abdominal fat tissue of various obese mouse models. Loss- and gain-of-function studies have established a protective role of visceral adipose tissue-associated Tregs in obesity-associated inflammation and metabolic abnormalities, presumably by inhibiting pro-inflammatory immune cells or stimulating activity of M2 macrophages [39]. A recent study demonstrated that the downregulation of KLF10 expression in CD4 + Tregs can lead to defective Treg function and subsequent altered glucose metabolism, obesity and insulin resistance [40]. Chronic infection in the pancreas causes not only direct damage to β cells, but can also induce autoimmunity especially in predisposed patients, which will enhance β cell destruction and reduce insulin production [37]. Immune integrity in the gastrointestinal tract was shown to exert important influence in pancreatic tolerance, where gut-primed Tregs can be potent mediators for the suppression of autoreactive T cells and promote tolerance to islet antigens, and enterovirus infection in young children is associated with depressed Treg function and increased inflammatory Th1/Th17 responses [41].

#### 3.2.2. Endothelial Dysfunction in Metabolic Syndrome

Endothelial function is affected by direct insult or indirect soluble mediators, which can promote clotting, vascular permeability and leucocyte recruitment. In the course of overwhelming or persistent activation, endothelial cells can be rendered dysfunctional, resulting in uncontrolled coagulation, vascular tone and permeability [26]. Endothelial dysfunction in metabolic syndrome can result from the adverse effect of oxidative stress, inflammatory cytokines, adipokines, hyperglycemia or free fatty acids, and it is the final common pathway between many cardiovascular risk factors and the development of atherosclerosis [4].

## 4. Essential Micronutrients in COVID-19 and Metabolic Syndrome

### 4.1. Vitamin D in COVID-19 and Metabolic Syndrome

Vitamin D is known to promote the generation of Tregs and support Treg suppressive function, and an inadequate amount can result in insufficient/dysfunctional Tregs [42]. Vitamin D can also induce DNA demethylation at vitamin D receptor (VDR) binding sites in dendritic cells and promote differentiation to tolerogenic dendritic cells with IL6-JAK-STAT3 pathway activation [43]. In addition to influencing Tregs, vitamin D was also shown to exert a direct effect on B cells, suppressing the differentiation of plasma cells and class switched memory B cells, thus modulating immunoglobulin production [44]. Hypovitaminosis D impairs mitochondrial functions and enhances systemic inflammation and oxidative stress. Vitamin D has antioxidant properties contributing to mitochondrial redox homeostasis [45]. Overall, dysregulated 1,25OHD plays a mechanistic and pathophysiological role in several disease processes that are shared with COVID-19, including diabetes, obesity and hypercoagulopathy [46].

#### 4.1.1. Benefits of Vitamin D in COVID-19

Vitamin D has attracted quite a lot of attention in COVID-19 because of its potential benefit of suppressing hyperinflammation, reducing cytokine storms and promoting better clinical outcomes. Several studies demonstrated associations of vitamin D with severe outcome and benefits of supplementing vitamin D in COVID-19. In a double-blind randomized controlled trial (RCT) of 321 recruited subjects, vitamin D supplementation in highly exposed individuals prevented SARS-CoV-2 infection without serious adverse reactions [47]. Another study of 228 older hospitalized patients showed that vitamin D supplementation was associated with lower severe COVID-19 (OR = 0.42, *p* = 0.0135) and ICU admission (OR = 0.341, *p* = 0.0076), thus supporting that vitamin D supplementation during the 3 months preceding the infection onset may have a protective effect on the development of severe COVID-19 [48]. A retrospective cohort of 15,968 patients that examined the relation of a prescription of a vitamin D metabolite given within 15–30 days before hospitalizations supported a beneficial association between prescription and patient survival for calcifediol (HR = 0.67, 95%CI 0.50–0.91) [49]. The GERIA-COVID quasi experimental study concluded that regular bolus vitamin D supplementation was associated with less severe COVID-19 and better survival in frail elderly [50]. The COVIT-TRIAL demonstrated that 6% of 127 patients allocated to high-dose cholecalciferol and 11% of 127 patients allocated to standard-dose cholecalciferol died within 14 days (adjusted hazard ratio = 0.39 (95% CI, 0.16 to 0.99, *p* = 0.049)) after controlling for randomization strata and baseline imbalances in important prognostic factors [51].

#### 4.1.2. Benefits of Vitamin D in Metabolic Syndrome

A cross-sectional study of 525 patients with type 2 diabetes versus 525 healthy controls indicated that a majority of patients (54.1%) had vitamin D deficiency, while 25.39% controls had hypovitaminosis D (*p* < 0.0001) [52]. A retrospective analysis of 36,523 adults from NHANES 2001–2014 demonstrated that each unit of decreased 25OHD concentration led to 0.025 higher HOMA-IR (insulin resistance assessed by homeostasis model assessment HOMA), thus a negative association between 25OHD and insulin resistance (IR) [53]. A meta-analysis of 29 randomized control studies (N = 3792) showed that oral supplementation of vitamin D had better effects in improving fasting blood glucose, HBA1c and fasting insulin compared with controls among prediabetics [54].

A prospective cohort of 37,079 patients with cardiovascular disease (CVD) from the UK Biobank study analyzed for the association of serum 25OHD with all-cause and cause-specific mortality demonstrated non-linear inverse associations for all-cause, cancer, respiratory disease and other-cause mortality (P-non-linearity < 0.01), with a 10 nmol/L increment in serum 25OHD concentrations associated with a 12% reduced risk for all-cause mortality and 9% reduced risk for CVD mortality [55]. Analysis of NHANES 2007–2014, which included 8799 participants, ≥20 yrs, studied the association of vitamin D and blood pressure: vitamin D was negatively related to systolic blood pressure but not to diastolic blood pressure, and magnesium intake improved the negative relationship of vitamin D and blood pressure, thus magnesium and vitamin D have an interactive effect in reducing systolic blood pressure [56]. A cohort study evaluated the association between changes in 25OHD and lipid levels among one cohort of individuals with increased vitamin D (group 1) versus decreased vitamin D (group 2) by >10 ng/mL over separate study periods, and each cohort showed that total cholesterol, low-density lipoprotein-C and triglyceride were decreased in group 1 but increased in group 2. These differences were significant after adjusting for age, sex, race, education, body mass index, blood pressure, smoking status, geographical location and baseline vitamin D and lipids (*p* < 0.001) [57].

#### 4.1.3. Vitamin D and Outcome of Vaccination

Vitamin D has been reported to be associated with the improved development of antigen-specific responses following vaccination. An experimental study demonstrated that vitamin D supplementation can boost antigen-specific immune response to cutaneous VZV antigen challenge in older adults with suboptimal vitamin D status [58]. However, studies on the effect of vitamin D in vaccination outcome in COVID-19 have generated conflicting results; some supported better vaccination outcome with higher vitamin D level, while others did not find any significant differences in SARS-CoV-2 IgG response as a function of vitamin D status [59,60]. Therefore, this issue remains open and further studies will be required for a more definitive answer.

### 4.2. Magnesium in COVID-19 and Metabolic Syndrome

Magnesium is involved as a co-factor in more than 300 enzyme systems and is required for fundamental processes such as energy production and nuclei acid synthesis. It is found in high concentration in mitochondria, is bound to ATP as magnesium-ATP and it is estimated that 3571 human proteins potentially bind to magnesium [61]. Magnesium is a natural calcium channel blocker, and extracellular magnesium concentration inhibits calcium entry into cells, which will affect calcium influx in smooth muscle cells and cardiomyocytes, in turn influencing the vascular tone [62]. Intracellular magnesium concentration has shown to be offset by calcium-related excitation–contraction coupling and decreases smooth cell responsiveness to depolarizing stimuli. In addition, magnesium administration may be useful in decreasing arterial blood pressure [63]. Magnesium diminished vascular tone and resistance by releasing NO, as well as antagonized the effect of vasoconstrictor molecules, e.g., calcium, bradykinin or angiotensin II [62]. Magnesium deficiency may promote endothelial cell dysfunction and increase the potential risk of thrombosis and atherosclerosis.

#### 4.2.1. Role of Magnesium in Chronic Inflammation

Low-grade inflammation (LGI) is an underlying factor in the pathological processes of many metabolic disorders. Chronic magnesium deficiency causes a reduction in extracellular magnesium and an increase in intracellular calcium concentration as well as priming of phagocytic cells and can promote chronic inflammatory responses. Inflammation affects pro-atherogenic changes in the metabolism of lipoproteins, affects endothelial dysfunction, influences host metabolism and is associated with increased reactive oxygen species, promoting membrane oxidation and NK-kB production [62]. It is widely accepted that an altered gut microbiota composition participates in systemic low-grade inflammation [64], and poor magnesium intake impairs intestinal absorption and promotes a pro-inflammatory response in overweight and obese subjects, which in turn impairs micronutrient absorption [62]. A comparative study showed that magnesium supplementation in obese subjects with or without type 2 diabetes affects microbial composition and functional potential [64]. A literature review of magnesium orotate and microbiome-gut–brain axis modulation showed that magnesium orotate is an important nutrient and adjuvant in the modulation of the microbiome-gut–brain axis and is emerging as a promising adjuvant therapy in dysbiosis and gut–brain axis modulation [65]. Furthermore, magnesium regulates gluconeogenic enzymes (including glucose-6-phosphatase) in the liver and exerts an anti-inflammatory effect in adipose tissue [62].

#### 4.2.2. Interaction between Magnesium and Insulin

Magnesium and insulin display a reciprocal relationship, with magnesium as a major factor determining insulin and glucose actions, while insulin in reverse regulates magnesium homeostasis [62]. Magnesium has a significant impact on insulin secretion and may contribute to dysfunction of pancreatic beta cells in type 2 diabetes. Insulin secretion is started by calcium influx that is competitively inhibited by extracellular magnesium, which acts as a calcium antagonist to inhibit its influx, and consequently the serum insulin level is inversely correlated with magnesium [64]. Conversely, physiological concentrations of insulin induce a specific increase in renal excretion of magnesium [66]. In fact, magnesium deficiency may not be just a secondary consequence, but patients with type 2 diabetes and hypomagnesemia actually enter a vicious circle in which hypomagnesemia causes insulin resistance and insulin resistance reduces serum magnesium concentrations [67]. 

#### 4.2.3. Interaction between Magnesium and Vitamin D

Magnesium is important for maximizing vitamin D function on different levels, including several steps in vitamin D metabolism which are dependent on magnesium as a co-factor, magnesium deficiency can decrease parathyroid hormone synthesis and secretion and also the number of available VDRs in target tissue, as well as magnesium deficiency is known to cause vitamin-D-resistant hypocalcemia, which can be corrected after magnesium replacement [68]. In a cross-sectional study, dietary magnesium intake influenced the associations of serum vitamin D levels with HOMA-β index and pancreatic β cell dysfunction [69]. Conversely, exogenous vitamin D increases magnesium absorption in the jejunum in healthy subjects, with vitamin D3 2000 IU/day for 6 months resulting in a significant increase in serum levels of magnesium (*p* < 0.04) [70]. Obese subjects are often magnesium and vitamin D deficient, and chronic latent magnesium deficiency and/or vitamin D deficiency predispose non-diabetic obese subjects to an increased risk of cardiometabolic diseases [64]. In a comparable study, patients given both magnesium and vitamin D experienced the greatest increase in serum 25OHD concentrations (6.3 +/− 8.36 ng/mL; *p* < 0.05), with a decrease in systolic blood pressure (7.5 +/− 8.26 mmHg; *p* < 0.05) for individuals who had a baseline systolic blood pressure >132 mmHg in this group. Therefore, a combined magnesium/vitamin D treatment may be more effective in increasing serum 25OHD concentrations compared with vitamin D supplementation alone in overweight/obese individuals [71].

#### 4.2.4. Limitation in Current Assessment of Magnesium Deficiency

For the assessment of magnesium adequacy, it is noteworthy that serum magnesium level may not be a reflective indicator of the total magnesium content in the body, because only 1% of magnesium is found in the extracellular fluid [72] and depletion of intracellular and/or ionized plasma magnesium can be found in individuals with normal total serum magnesium [73]. Therefore, normal serum magnesium does not rule out moderate or severe magnesium deficiency, and instead we may need to consider measuring ionized, red blood cell or tissue magnesium [74]. A comparative study has shown that both ionized magnesium and intracellular magnesium but not serum total magnesium were significantly reduced in type 2 diabetes compared with non-diabetic control subjects [75].

#### 4.2.5. Benefits of Magnesium in COVID-19

Magnesium supplementation can offer multiple potential benefits in COVID-19, including a strong vasodilator and bronchodilator effect [76] and reducing low-grade persistent inflammation, endothelial dysfunction and thrombotic risk [77]. However, there were only limited studies to assess the impact of magnesium in COVID-19, but they generally supported a beneficial role. A retrospective cohort study that reviewed 83 patients hospitalized for COVID-19 demonstrated that hypomagnesemia may increase symptoms associated with mortality in COVID-19 [78]. Another cross-sectional study showed that a higher intake of dietary magnesium is inversely associated with COVID-19 severity and symptoms with lower odds of severe COVID-19 (OR = 0.32; 95% CI 0.15–0.70) [79]. A study based on the understanding of the requirement of TMPRSS2 expression for viral entry in SARS-CoV-2 gave magnesium to patients for 12 weeks, which modified the TMPRSS2 phenotype, indicative of possible a role in intervention for COVID-19 [80].

#### 4.2.6. Benefits of Magnesium in Metabolic Syndrome

The high prevalence of hypomagnesemia has been reported in subjects with type 2 diabetes, which was mainly caused by a low intake and increased urinary loss. A large Chinese population study reported an inverse correlation between dietary magnesium intake and prevalence of metabolic syndrome, and magnesium is inversely associated with the risk of type 2 diabetes in a dose–response manner [64]. A cross-sectional study measuring serum ionized magnesium in 290 T2DM patients demonstrated 49.3% with hypomagnesemia at a cutoff of magnesium <0.46 mmol/L [81]. A review of the literature of magnesium in type 2 diabetes estimated an incidence of 13.5 to 47.7% of type 2 diabetes with hypomagnesemia linked to poor glycemic control, coronary artery disease, diabetic retinopathy, nephropathy and neuropathy [82]. A case–control study of 4447 participants demonstrated an independent and inverse association between plasma magnesium with prediabetes and type 2 diabetes in a Chinese population. ORs from the lowest to highest quartiles of plasma magnesium were 1, 0.57(95%CI, 0.44–0.74), 0.49(0.37–0.65) and 0.51(0.37–0.70) for prediabetes; ORs from the lowest to highest quartiles of plasma magnesium were 1, 0.26(0.20–0.33), 0.15(0.12–0.20) and 0.15(0.11–0.20) for type 2 diabetes [83]. A systematic review of 10 studies showed that circulating magnesium levels in people with prediabetes were significantly lower than healthy controls (Weighted Mean Difference, WMD = −0.07 mmol/L; 95% CI −0.09,0.05 mmol/L, *p* < 0.001) [84].

A meta-analysis of 12 articles supported the beneficial effect of magnesium supplementation in reducing insulin resistance in patients with hypomagnesemia presenting insulin resistance [85]. A systematic analysis of 18 RCTs supported modest improvement of fasting blood glucose and HbA1c with oral magnesium supplementation [86]. In a 7-year follow-up study of 2582 participants, the highest magnesium intake had a 37% lower risk of incident metabolic impairment (*p* = 0.02), and in those with baseline metabolic impairment, higher intake was associated with a 32% lower risk of incident diabetes (*p* = 0.05) [87]. A meta-regression analysis of 25 prospective cohort studies including 22,828 subjects with type 2 diabetes showed that after adjusting for age and body mass index, the risk of type 2 diabetes incidence was smaller by 8–13% for a 100 mg magnesium increment in intake per day. A meta-analysis of nine articles and 31,876 subjects demonstrated that a higher magnesium intake is associated with a lower risk of metabolic syndrome (OR = 0.73, 95% CI 0.62–0.86, *p* < 0.001) [62]. A randomized double-blind, placebo-controlled trial of 60 subjects suffering from coronary heart disease and type 2 diabetes, with the treatment arm given magnesium 250 mg/Zn 150 mg for 12 weeks versus the control arm taking placebo, showed beneficial effects of decreased fasting plasma glucose (β −9.44 mg/dL, 95% CI −18.30, −0.57, *p* = 0.03) and insulin levels (β −1.37 uIU/mL, 95% CI −2.57, −0.18, *p* = 0.02) when co-supplemented [88]. A prospective longitudinal 20-year study of 4497 adults without diabetes at baseline demonstrated that the multivariable adjusted hazard ratio (HR) of diabetes in the highest quartile of magnesium intake was 0.53 (95% CI, 0.32–0.86, *p* < 0.01) compared to those in lowest quartile, magnesium intake was significantly inversely associated with IL6, fibrinogen and HOMA-IR, magnesium serum level was inversely correlated with HOMA-IR, and magnesium intake was inversely longitudinally associated with incidence of diabetes, explained in part by the inverse correlation of magnesium intake with systemic inflammation and insulin resistance [89].

A study of 2362 subjects without CVD at baseline demonstrated a 34% lower risk (95% CI: 0.51–0.87) of CVD compared to a magnesium intake of ≥320 vs. <240 mg/day [90]. A systematic review of nine articles including 20,119 cases of hypertension showed that a 100 mg/day increment in magnesium intake was associated with a 5% reduction in the risk of hypertension (RR = 0.95, 95%CI 0.90–1.00). A meta-analysis found that magnesium significantly reduces systolic blood pressure and diastolic blood pressure along with increases in serum magnesium concentration. Several studies have demonstrated the preventive effect of increased dietary magnesium supply on dyslipidemia, which may be linked to the fact that magnesium participates in the modulation of lipoprotein lipase (LPL), desaturase (DS), HMG-CoA reductase and lecithin-cholesterol acyl transferase (LCAT). In the analysis of 1999–2002 NHANES, especially in subgroups of overweight/obese subjects of age > 40 yrs, those consuming magnesium at less than 50% recommended daily allowance were 2.24 times more likely to have elevated C-reactive protein that adults with proper magnesium intake [62].

### 4.3. Vitamin B in COVID-19 and Metabolic Syndrome

B vitamins are synthesized by plants, yeasts and bacteria but not by mammals, so mammals must acquire B vitamins from dietary or commensal bacteria in their intestines, and therefore the composition and function of the intestinal microbiota may affect host B vitamin usage. Commensal bacteria are both providers and consumers of vitamin B, and there may be competition between the host and intestinal microbiota for vitamin B [91]. Vitamin B12 also acts as a modulator of gut microbiota, and low levels can result in increased inflammation, reactive oxygen species and oxidative stress [92]. Vitamin B performs many important functions in the distal colon: (a) a nutrient for host and microbiota, (b) a regulator of immune cell activity, (c) a supporter of the survival of certain bacterium and (d) a suppressor of colonization of pathogenic bacteria [93]. The B vitamin complex is essential in supporting mitochondrial function, especially as a nutritional co-factor or coenzyme that is located in mitochondria [94]. Various vitamin B subunits are important for proper immune function, e.g., a vitamin-B1-deficient diet showed impaired maintenance of naïve B cells; vitamin B3 can inhibit the production of pro-inflammatory cytokines IL-1, IL-6 and TNF-a by macrophages and monocytes; vitamin B9 is a survival factor for Treg cells and is required for Treg maintenance; and vitamin B12 deficiency decreases the number of CD8 + T cells and suppresses natural killer T cell activity in mice [91].

#### 4.3.1. Importance of Vitamin B in Gut Dysbiosis

Vitamin B12 impacts diverse host–microbe symbiosis and may make a contribution in shaping the structure and function of human gut microbial communities [95]. Studies conducted on twins have revealed differences on phyla distribution between obese and lean subjects, e.g., relative reductions in Bacteroidetes and Actinobacteria were found among obese subjects, and specific changes in gut microbiota composition could be identified in each progressive stage leading to the development of diabetes [96]. In dysbiosis conditions, e.g., a low Bacteroidetes to Firmicutes ratio, there is high level of lipopolysaccharides and pro-inflammatory cytokines, and vitamin B12 produced by resident bacteria in the colon is important to regulate gene expression in gut Bacteroidetes [97].

#### 4.3.2. Importance of Vitamin B in COVID-19

A few independent studies demonstrated the potential direct antiviral effect of vitamin B12 against SAR-CoV-2. A study deploying a quadratic unbound binary optimization (QUBO) model to search for other antiviral compounds similar to remdesivir that found efficacy of vitamin B12 against several variants, including the alpha, beta and delta of SARS-CoV-2, suggested that vitamin B12 may be considered as a SARS-CoV-2 antiviral candidate [98]. Another study using a computational approach found significant binding of vitamin B12 with furin, RNA-dependent RNA polymerase, Mpro, ORF3a and ORF7a, which may render vitamin B12 a suitable candidate to reduce the virulence of SAR-CoV-2 [99]. A quantitative high-throughput screening also identified vitamin B12 as an effective SARS-CoV-2 3CL protease inhibitor [100]. A case–control matched cohort study on COVID-19, with a first cohort of 6202 hospitalized patients with 5 controls matched to each case, and second cohort of 6919 non-hospitalized patients with 2 controls matched to each case, demonstrated significant reduction in the odds for hospitalization for COVID-19 when treated with vitamin D, magnesium, vitamin B12 or calcium-zinc [101].

#### 4.3.3. Importance of Vitamin B in Metabolic Syndrome

Vitamin B12 deficiency has been demonstrated to be prevalent among patients with type 2 diabetes. However, B12 insufficiency was significantly lower in 117 metformin users (18.4%) compared with 80 non-metformin users (27.9%), and B12 deficiency was significantly higher in 25 metformin users (3.9%) compared with 6 non-metformin users (2.1%) [102]. A double-blind RCT of administering folate and vitamin B12 improved insulin resistance and endothelial function along with decreasing homocysteine levels [103]. A cross-sectional study of NHANES 2011–2014, with 9075 participants ≥20 yrs old, showed that the multivariable adjusted odds ratios (95% CIs) of obesity were 1.00 (reference), 0.95(0.79,1.14), 0.86(0.74,0.99) and 0.71(0.60,0.84) (*p* for trend <0.001) for increasing quartiles of serum vitamin B12 concentrations, higher serum vitamin B12 was inversely associated with obesity, low vitamin B12 was associated with increased insulin resistance in adult patients and obese adolescents and low vitamin B12 and high tHcy were associated with higher risk of all-cause and CVD deaths in aged women [104]. Vitamin B12 supplementation reduced the risk of stroke in patients with CVD and/or renal diseases [105]. Both folate and vitamin B12 have a synergistic effect on the markers of obesity and lipid profiles, including waist to hip ratio, triglyceride and high-density lipoprotein levels [106].

## 5. Putting Everything All Together

It is noteworthy that deregulated immunity, endothelial dysfunction and gut dysbiosis, which are associated with severe outcome in COVID-19, are essentially the same factors which are involved in the development of metabolic syndrome. In addition, the same micronutrients which had demonstrated benefits in COVID-19 and metabolic syndrome, eg. vitamin D, magnesium and vitamin B, are also implicated in the proper function of immune regulation and endothelial and gut tissue. Together they seem to imply a close relationship between COVID-19 and metabolic syndrome, and in fact, COVID-19 may represent a prototype of an acquired etiological agent for metabolic syndrome.

**Figure 1:**
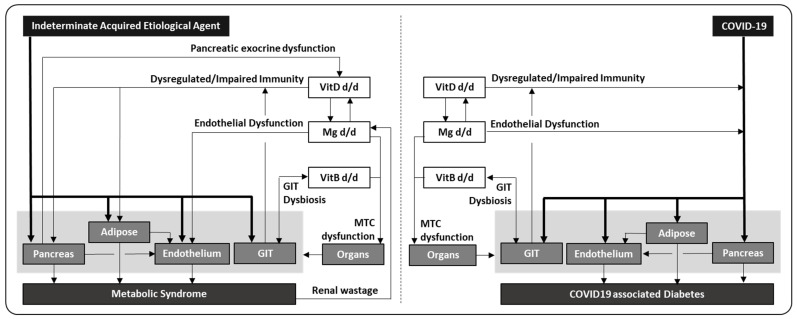
Comparison between classical and COVID-19-associated metabolic syndrome. In COVID-19, SARS-CoV-2 can infect pancreas and adipose tissue directly, leading to impaired insulin production and insulin resistance, while endothelium and gut involvement can result in immunothrombosis and microbial dysbiosis. Concomitant deficiency in essential micronutrients will aggravate COVID-19, e.g., hypovitaminosis D causing deregulated immune response and hyperinflammatory tissue damages, hypomagnesemia causing endothelial dysfunction and excessive thrombosis, as well as hypovitaminosis B causing gut dysbiosis and impaired immunity. In addition, both magnesium and vitamin B deficiency can affect mitochondrial function, further aggravating insulin resistance and hyperglycemia. Together, these tissue dysfunctions and micronutrient deficiencies play an important role in contributing to the development of COVID-19-associated diabetes mellitus (**right panel**). In metabolic syndrome, patients can present with deranged function in pancreas, adipose, endothelial and gut tissue, but the exact etiological agent and early pathological events are generally indeterminate at the time of diagnosis. Vitamin D, magnesium and vitamin B deficiencies are common in these patients, which are both a consequence of the deranged metabolic state as well as contributing factors in sustaining the metabolic derangement. This vicious cycle may ultimately spiral downwards and culminate in classical metabolic syndrome if it remains unabated. In comparison to COVID-19, it is evident that the important tissue dysfunctions and micronutrient deficiencies are similar in both diseases, though a self-sustaining vicious cycle has been demonstrated mainly in classical metabolic syndrome. Therefore, SARS-CoV-2 may serve as a prototype of an acquired etiological agent, and COVID-19-associated diabetes may provide the opportunity to understand the missing initial factors and events, in the development of classical metabolic syndrome (**left panel**).

SAR-CoV-2 demonstrates high tropism to enter vascular space, which will enhance its spread to multiple organs, and infecting pancreas and adipose tissue can induce metabolic derangement and result in impaired insulin production and insulin resistance. The pro-inflammatory milieu can also stimulate gluconeogenesis [107] causing hyperglycemia, but some patients may remain euglycemic due to adequate early compensatory hyperinsulinemia [11]. Subsequently, the altered glucose and lipid profiles can affect endothelium adversely, and all together initiate a disturbed metabolic state. These impairments can be further aggravated in patients with deficiency in essential micronutrients, e.g., vitamin D deficiency can result in immune dysregulation, driving hyper-inflammation and excessive end-organ damages; magnesium deficiency can negatively affect endothelial function, resulting in increased vascular tension with thrombotic risk; vitamin B deficiency causing gut dysbiosis can affect micronutrient absorption/utility with downstream impaired immunity; and combined vitamin B/magnesium deficiency can affect mitochondrial function adversely and worsen pancreatic and adipose functions. Since vitamin D, magnesium or vitamin B deficiency is relatively common in diabetic patients, it corroborates with the worst outcome observed in COVID-19 patients with concurrent metabolic syndrome (Figure 1, right panel). If any initial infective driver persists and develops into chronic infection or converts to immune complication, it will sustain both insulin resistance and higher glucose production, prolonging the presence of hyperinsulinemia. In turn, hyperinsulinemia can reduce the resorption of magnesium in renal tubules, resulting in hypomagnesemia and consequently or directly impairing mitochondrial function, which in reverse aggravates insulin resistance and overall cellular metabolism and probably marks the transition from localized to systemic insulin resistance. When these derangements begin progressing to chronic relative hyperinsulinemia with paradoxical hyperglucagonemia, pancreatic insufficiency and adipose dysfunction, they can potentially establish a vicious cycle, and the final outcome is a downward spiral in insulin production, insulin resistance, hyperglycemia and dyslipidemia, which eventually evolves into classical metabolic syndrome (Figure 1, left panel). However, it is important to note that genetic variants which can independently influence the immune/endothelial/mitochondrial functions, and the absorption/retention of essential micronutrients, are not included in this schema.

The COVID-19 pandemic is indeed a unique opportunity that may provide a glimpse of possible early events which are generally not readily accessible, in the initial establishment of metabolic syndrome. The proposed parallel also postulates the possibility that multiple deficiencies and dysfunctions observed in metabolic syndrome may not be just a consequence; instead, they are contributing to the overall development. This raises important implications in the management of metabolic syndrome, where appropriate antiviral/antimicrobials may need to be considered to eradicate suspect microbes and consequential chronic inflammation, while repleting essential micronutrients can reverse immune-vasculopathy to reduce damage to pancreas, adipose and gastrointestinal tissues, as well as optimize mitochondrial function to maximize overall cellular metabolism and reverse insulin resistance. All these additional measures are in fact complementary to the current effort to optimize insulin availability, minimize the adverse effect of excessive gluconeogenesis and reduce hyperglycemic damages in many end organs. In addition to targeting the specific microbes, administration of probiotics to improve the gut microbiome should be studied to ascertain its full benefit. The list of micronutrients discussed above is not comprehensive but includes mainly the archetype for each specific pathogenic component, thus many other micronutrients, e.g., fat-soluble vitamins A or E, bivalent metals calcium, manganese and zinc, and water-soluble vitamin C should also be studied for their role in maintaining a healthy immunoendothelial, gastrointestinal and mitochondrial system. Other than micronutrients, essential biomolecules which play a role in mitochondrial function also warrant attention, e.g., melatonin, which is essential to optimize mitochondrial function, may be reduced in production when tryptophan is redirected to kynurenine instead of the serotonin pathway under the influence of inflammatory signals [108,109].

Conversely for COVID-19 management, the primary focus to eradicate SARS-CoV may need to be augmented with the restoration of normal mitochondrial, immunoendothelial and gastrointestinal functions. They are essential not only for improvement of overall outcome in a single infection, but also to prevent cumulative damages from repeated inflammation and hypercoagulopathy with every subsequent episode of re-infection. This strategy may also prepare us in the future not just to handle SARS-CoV-2 but other infectious etiologies which are dependent on similar combinations of dysfunctions to produce such significant morbidity and mortality. Furthermore, administrating vaccination to COVID-19 patients may need to be mindful of the underlying metabolic status, because it may help to prevent development of new metabolic disease when given early in patients with adequate residual pancreatic function, however, it may potentially aggravate the metabolic impairment in patients with chronic metabolic syndrome, especially when the immune response is excessive with pancreatic involvement.

Finally, this discussion is not intended to provide a comprehensive comparison between COVID-19 versus classical metabolic syndrome, but rather to highlight parallels in both disorders and to appraise a possible common underlying pathophysiology. If this expanded appreciation of metabolic syndrome can indeed be translated to a more efficacious therapeutic strategy, peradventure maybe even offering a curative option beyond the current focus on reversing downstream complications, it is our sincerest hope that it may bring some solace to those who had suffered such a great loss in this unprecedented time, at least knowing that all their bereavement may not be in vain, but has instead contributed to the improvement in the general health of all humankind.

## Data Availability

Not applicable.

## Abbreviations

VitD = vitamin D, Mg = magnesium, VitB = vitamin B, d/d = dysfunction/deficiency, GIT = gastrointestinal tract, MTC = mitochondrial.
